# Praziquantel resistance in schistosomes: a brief report

**DOI:** 10.3389/fpara.2024.1471451

**Published:** 2024-10-02

**Authors:** Gabriela Eastham, Dane Fausnacht, Matthew H. Becker, Alan Gillen, William Moore

**Affiliations:** ^1^ Department of Biology and Chemistry, School of Health Sciences, Liberty University, Lynchburg, VA, United States; ^2^ Department of Biology, School of Sciences and Agriculture, Ferrum College, Ferrum, VA, United States

**Keywords:** schistosomiasis, praziquantel, resistance, efficacy, mass drug administration

## Abstract

Schistosomiasis is a group of both acute and chronic parasitic trematode infections of the genus *Schistosoma*. Research into schistosomiasis has been minimal, leading to its classification as a neglected tropical disease, yet more than 140 million people are infected with schistosomes globally. There are no treatments available for early-stage infections, schistosomal dermatitis, or Katayama syndrome, other than symptomatic control with steroids and antihistamines, as the maturing organisms seem to be mostly resistant to typical antiparasitics. However, praziquantel (PZQ) has been the drug of choice for schistosomiasis for decades in the latter stages of the disease. Though it is effective against all three clinically relevant species, heavy reliance on PZQ has led to concerns of schistosome resistance, especially in areas that have implemented this drug in mass drug administration (MDA) programs. This article summarizes the available literature concerning the available evidence for and against a warranted concern for PZQ resistance, genomic studies in schistosomes, proposed mechanisms of resistance, and future research in alternative methods of schistosomiasis treatment.

## 1 Introduction

Drug resistance is a well-known and key phenomenon that has impacted the effectiveness of medications such as some antibiotics and antihelminthics for treating poultry, livestock, and humans (Mohamed et al., 2022; Malik et al., 2022; Qamar and Alkheraije, 2023; Li et al., 2023). Resistance is defined as a significant increase in the frequency and unresponsiveness of individuals in a susceptible population to a compound (Prichard et al., 1980 *;* Coles and Kinoti, 1997; Greenberg, 2013). Unlike tolerance, resistance is heritable and due to a population’s previous drug exposure (Prichard et al., 1980; Greenberg, 2013).

**Figure 1:**
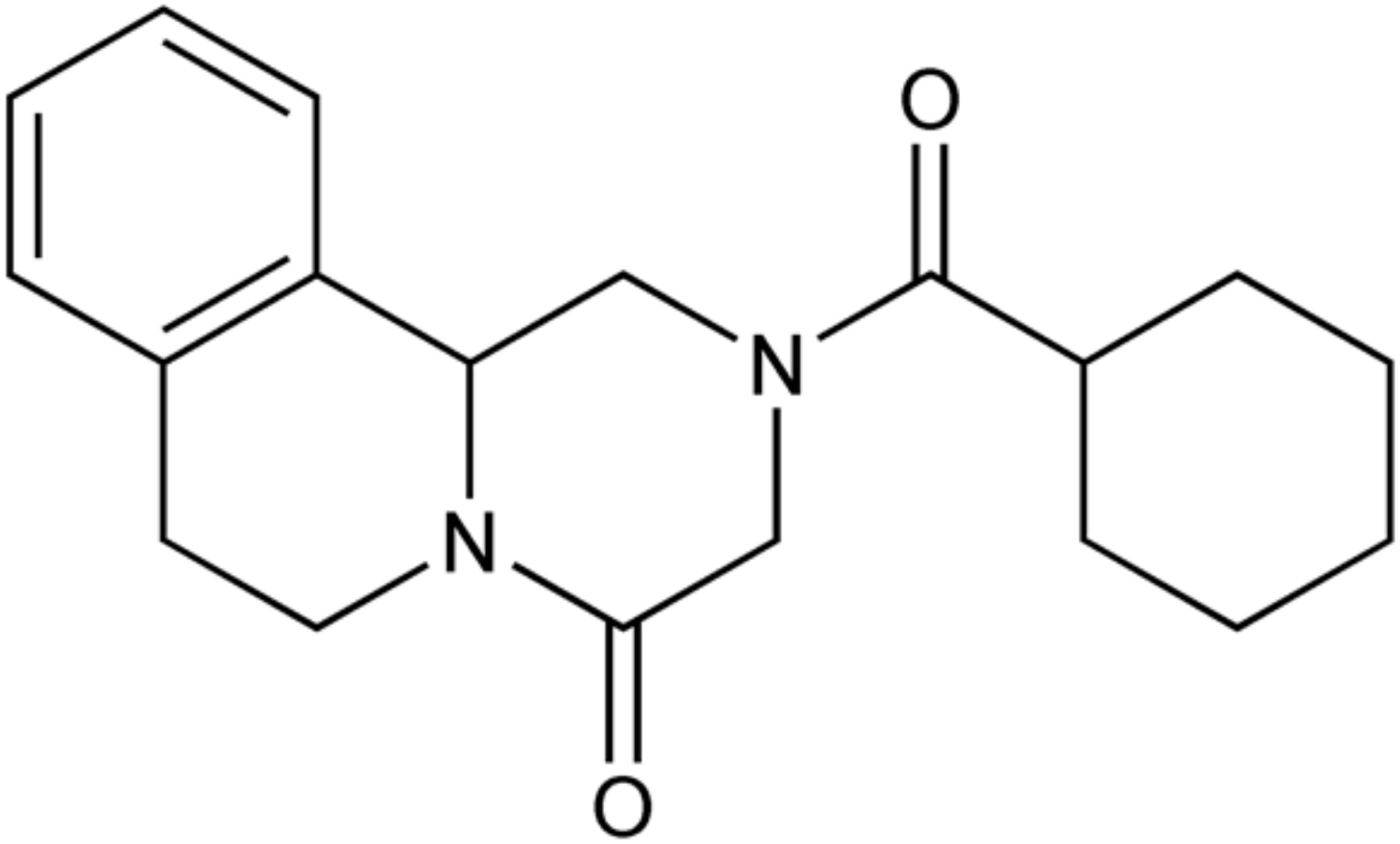
Chemical structure of praziquantel (Royal Society of Chemistry, 2024).

Emerging drug resistance against the broad-spectrum antihelminthic drug praziquantel (PZQ) (**Figure 1**) has been a growing public health concern (Berger et al., 2021; Cotton and Doyle, 2022). There has been much discussion as to whether PZQ resistance is imminent or widespread (Danso-Appiah and De Vlas, 2002; Botros et al., 2005; Melman et al., 2009; Crellen et al., 2016; Fukushige et al., 2021). Following its discovery in the 1970s by the pharmaceutical companies Merck and Bayer, PZQ has become the drug of choice to treat schistosomiasis, the second most debilitating tropical disease after malaria (Abdel Aziz et al., 2022). The causative schistosomes, or blood flukes, are also responsible for approximately 200,000 annual deaths and affect over 250 million people globally, making them the most important helminthic infectious agents (World Health Organization, 2021).

Schistosomiasis is caused by three main species: *Schistosoma mansoni* is the most widespread and is the only species known to exist in the Western Hemisphere, *S. haematobium* is found in Africa and the Middle East, and *S. japonicum* is found in regions of Southeast Asia (World Health Organization, 2023a). Schistosomes have a complex life cycle that involves two hosts—a mammalian definitive host and a freshwater snail from the genus *Biomphalaria* as an intermediate host. Schistosoma eggs are released from either the feces or urine of an infected individual, depending on the species of schistosome (Centers for Disease Control and Prevention, 2024). The larvae, called miracidia, infect the suitable snail host for two generations to produce free-swimming larval cercariae. These cercariae then shed their tails, penetrate the skin of the mammalian host, and become schistosomulae (Centers for Disease Control and Prevention, 2024). Following infection, the schistosomulae mature and undergo sexual reproduction. The adult females lay 300 – 3,000 eggs daily until the end of the worm’s lifespan—about three to five years (Cahill, 2011). Some of the eggs build up in the host’s tissue, causing inflammation and a host immune response that results in the disease’s morbidities (Burke et al., 2009; Colley et al., 2014). Other eggs are passed through the intestinal or bladder mucosa and are expelled in the feces or urine, completing the cycle (Cahill, 2011).

In endemic areas, MDA of PZQ is essential for schistosomiasis control as a form of preventative chemotherapy due to the drug’s affordability, availability, minimal side effects, and effectiveness against human infections of trematodes and cestodes (Cioli et al., 2014; Crellen et al., 2016; Nogueira et al., 2022; Villamizar-Monsalve et al., 2024). This extensive use has raised concerns about emerging drug resistance, which may develop following prolonged and repeated application, such as in MDA (Coles and Kinoti, 1997; Geerts et al., 1997).

Laboratory-induced resistance to PZQ have been successful, and there have been multiple reports of reduced PZQ efficacy in the field following continuous drug exposure (Ismail et al., 1994; Fallon, 1998; Ismail et al., 1999; Melman et al., 2009; Couto et al., 2011; Li et al., 2011; Lotfy et al., 2015; Crellen et al., 2016). However, it has also been observed that reduced PZQ sensitivity characteristics have dissipated after several generations, even in the presence of drug pressure (Botros et al., 2005; Melman et al., 2009). In addition, multiple areas have endured several years of PZQ treatment, and the efficacy rates remain high (Seto et al., 2011; Mduluza et al., 2020; Tetteh-Quarcoo et al., 2020).

This review draws together previous and recent literature about PZQ efficacy across the three main schistosome species in the context of emerging PZQ resistance. Brief updates on the PZQ mechanism of action, proposed mechanism of resistance, impacts of MDA on schistosome genetic diversity, PZQ alternatives, and schistosomiasis vaccine development are also covered.

## 2 Praziquantel efficacy

PZQ treatment efficacy is commonly measured by the egg reduction rate (ERR), which compares the pre-treatment and post-treatment number of eggs shed in the urine or feces. Another prevalent method is the cure rate (CR), which compares the number of infected individuals who become negative for schistosomiasis post-treatment (Fukushige et al., 2021).

Due to its effectiveness as an antiparasitic, PZQ is on the WHO’s list of essential medicines, and in 2017, approximately 100 million individuals received PZQ treatment for schistosomiasis (Park and Marchant, 2020). However, PZQ is less effective against juvenile worms or schistosomulae (Pica-Mattoccia and Cioli, 2004; Villamizar-Monsalve et al., 2024). It is thought that ATP-binding cassette (ABC) transporters, which can export toxins, play a role in this protection, as juvenile worms have about two and a half times the number of ABC transporters as the adult form (Kasinathan et al., 2010). In addition, PZQ must be administered in higher than recommended doses to efficiently kill schistosome eggs (Richards et al., 1989). Therefore, a follow-up dose 4 to 6 weeks after the initial dose may be necessary to prevent reinfection after any juvenile worms have matured (Gryseels et al., 2006).

### 2.1 Praziquantel mechanism of action and mechanism of resistance

Despite being the drug of choice against helminth infections for decades, the exact mechanism of action for PZQ is unclear. In trematodes and cestodes, PZQ may activate the transient receptor potential ion channel in the worm (Sm.TRPM_PZQ_) by engaging with a hydrophobic ligand-binding pocket, which opens the voltage-gated Ca^2+^ channels and pumps (Park and Marchant, 2020). This causes membrane depolarization, which is followed by rapid, involuntary tetanic muscle contractions and paralysis of the worm (Nogueira et al., 2022). PZQ may also change or destroy the worm’s integument, exposing its previously hidden parasitic antigens and leaving it vulnerable to host immune defenses (Eyoh et al., 2019).

Although a mechanism of PZQ resistance has also not been well characterized, it has been suggested that resistant worms are simply better able to metabolize the drug compared to non-resistant worms (Zdesenko and Mutapi, 2020). According to a recent study using whole-genome sequencing, it is also possible that genetic variation at or near the Sm.TRPM_PZQ_ channel could be involved (Le Clec’h et al., 2021). However, further research on wild-type schistosomes and their Sm.TRPM_PZQ_ ion channels across a variety of regions is needed for more conclusive answers.

## 3 Praziquantel resistance

Several laboratory studies have successfully induced PZQ resistance in schistosomes, particularly *S. mansoni*. In 1994, an *in vitro* study subjected a population of *S. mansoni*-infected mice to increasing PZQ drug pressure. By the seventh generation, 93% of the resistant schistosomes survived three PZQ doses of 300 mg/kg, which killed 89% of the control group (Fallon, 1998; Vale et al., 2017). Another study showed that resistance to the therapeutic dose of PZQ can be induced in following generations of *S. mansoni* in mice through successive subcurative doses (Ismail et al., 1994). A simpler and less expensive technique was later developed to induce PZQ resistance in *S. mansoni* through successively treating infected *Biomphalaria glabrata* snails with 100 mg/kg of PZQ (Couto et al., 2011). More recently, a study in 2015 induced resistance in an Egyptian strain of *S. mansoni* through treating multiple subcurative doses of *Biomphalaria alexandrina* snails (Lotfy et al., 2015). Regarding *S. japonicum*, an unsuccessful attempt was made in 1990 to experimentally induce resistance using drug pressure through infected mice (Yue et al., 1990). Induction of resistance in *S. japonicum* in the three life stages—adult, cercaria, and miracidia— was later achieved (Li et al., 2011). These studies demonstrate that schistosomes are capable of developing resistance under PZQ drug pressure of subcurative doses. There is currently no knowledge of laboratory-induced resistance to PZQ in *S. haematobium*.

The first significant instance of reported PZQ resistance on the field occurred in 1994 during an *S. mansoni* infection outbreak in Senegal (Stelma et al., 1995). The standard single-dose treatment of 40 mg/kg resulted in alarmingly low cure rates of 18-36% rather than the usual 60-90% (Doenhoff et al., 2008; Stelma et al., 1995). However, a 20 mg/kg dose of oxamniquine, an alternative anthelminthic drug, showed a typical cure rate (Stelma et al., 1997). It has been suggested that the low cure rate of PZQ was due to an intense initial infection, as the average egg counts were notably high in patients (Stelma et al., 1995; Utzinger et al., 2000; Danso-Appiah and De Vlas, 2002). PZQ is less effective against juvenile schistosomes and eggs, which may have survived the initial treatment and matured into adults after treatment (Richards et al., 1989; Danso-Appiah and De Vlas, 2002; Pica-Mattoccia and Cioli, 2004).

Another early report of apparent *S. mansoni* field resistance to PZQ was in villages in the Nile Delta region of Egypt (Ismail et al., 1996). All patients were treated with the standard dose of 40 mg/kg followed by a second 40 mg/kg dose or a third 60 mg/kg dose if necessary (Ismail et al., 1996). Although the PZQ cure rate was 98.4%, eggs isolated from the uncured patients generated PZQ infections in mice that were 3-5 times less sensitive to PZQ, raising concerns about resistance to PZQ in the parent worms (Ismail et al., 1996, 1999). When treated with PZQ *in vitro*, the isolates from the uncured patients showed decreased muscle contraction, decreased tegumental disruption, and decreased calcium influx, all of which are well-characterized PZQ actions on schistosomes (Ismail et al., 1999; William et al., 2001; William and Botros, 2004; Eyoh et al., 2019; Nogueira et al., 2022). Although these results were concerning, a study conducted in the same villages using the same dosing regimen ten years after the initial studies revealed no resistance to PZQ despite a decade of continued and broad use of the drug. It is worth noting that the infections initially present for the follow-up study were generally light. In addition, detecting a change from the previous 98.4% cure rate was not possible due to the sample size (Botros et al., 2005).

Reduced sensitivity to PZQ was later reported in Kenya among isolates of *S. mansoni* gathered from patients who had been previously treated with PZQ but whose occupations continuously exposed them to infection. The study also analyzed an isolate from a patient who had been treated with PZQ 18 times and never fully cured (KCW). This isolate was significantly less susceptible to PZQ both *in vivo* and *in vitro*. However, one KCW sub-isolate retained its resistant characteristics through 6 generations without any PZQ treatment. Meanwhile, another KCW sub-isolate returned to PZQ sensitivity after retesting for 8 generations. Such an occurrence may inform the results of the Nile Delta villages studies, suggesting that reduced PZQ susceptibility is not a stable trait in schistosomes and may require a biological cost (Botros et al., 2005; Melman et al., 2009; Greenberg, 2013).

A repeated cross-sectional study in Uganda found statistically reduced PZQ efficacy against *S. mansoni* among children from schools that had received 8 or 9 rounds of mass drug administration (MDA) than children from schools that had received 5 rounds or 1 round. Interestingly, a whole-genome sequencing study of the miracidia collected revealed that genomic diversity remained varied and unstructured despite long-term PZQ use. Therefore, the previously reported low PZQ efficacy may have been due to factors other than resistance (Crellen et al., 2016; Berger et al., 2021).

Concern for PZQ resistance in wild-type *S. japonicum* had received discussion due to its heavy use in endemic areas of China (Yu et al., 2001; Wu et al., 2011; Wang et al., 2012). Field studies have tested the efficacy of PZQ to *S. japonicum* in areas of varying endemicities throughout China using a single oral dose of 40 mg/kg (Liang et al., 2001; Wang et al., 2012). The results suggest that despite thirty years of heavy and expanded chemotherapy, sensitivity to PZQ in *S. japonicum* has not significantly decreased in China (Liang et al., 2001; Wang et al., 2012). In another study, the efficacy of PZQ against *S. japonicum* was compared between an area with repeated PZQ chemotherapy and a newly identified endemic area. The results indicated that the efficacy between the two areas were not significantly different (Yu et al., 2001). A cross-sectional study across 33 villages in Sichuan Province was organized to evaluate PZQ efficacy against *S. japonicum* (Seto et al., 2011). Out of 185 cases, only one remained uncured after receiving two doses of 40 mg/kg of PZQ, indicating that PZQ remains an effective treatment for *S. japnonicum* in China (Seto et al., 2011).

Regarding *S. haebatobium*, a recent study in Ghana detected no sign of its resistance to PZQ and attributed the more persistent schistosomiasis cases to reinfection (Tetteh-Quarcoo et al., 2020). Occasionally, there have been isolated reports of failed standard treatment of *S. haematobium* in travelers returning from endemic areas (Herwaldt et al., 1995; da Silva et al., 2005; Alonso et al., 2006). Various possible explanations exist for these instances, including the presence of a concurrent infection and the therapeutic failure of a single 40 mg/kg dose of PZQ (Herwaldt et al., 1995; da Silva et al., 2005). Since PZQ acts in synergy with the host immune system, it has been hypothesized that some individuals originating from non-endemic areas may lack the necessary immunological factor to overcome the infection (Wu et al., 2011; Vale et al., 2017).

A meta-analysis and systematic review article in 2023 have reported that PZQ efficacy has remained high, and there is no consistent evidence for the emergence of PZQ resistance in schistosomes (Fukushige et al., 2021; Aboagye and Addison, 2023). However, care should be taken to attempt to prevent schistosome resistance on the field, such as avoiding treatment with subcurative doses of PZQ, as this has been shown to experimentally induce resistance in *S. mansoni* and *S. japonicum* (Fallon, 1998; Li et al., 2011; Wang et al., 2012). Focus should also be placed on alternative methods of schistosomiasis control, such as snail control, clean tap water, health education, and building latrines (Wang et al., 2012; Villamizar-Monsalve et al., 2024). In addition, drug quality should continue to be monitored to ensure the effectiveness of praziquantel and detect further cases of suspected resistance (Wang et al., 2012; World Health Organization, 2022).

### 3.1 Continued use of praziquantel

Despite its ineffectiveness against juvenile schistosomes, inability to prevent reinfection, and heavy discussion of schistosome resistance, PZQ will remain the drug of choice for schistosomiasis for the foreseeable future. After decades of constant use, efficacy rates remain high and incidences of resistance are rare (Fukushige et al., 2021; World Health Organization, 2022). In 2022, the WHO published guidelines on the control and elimination of schistosomiasis in humans which recommended continued and expanded access to PZQ (World Health Organization, 2022).

### 3.2 Genetic diversity of schistosomes

The genetic consequences of MDA and drug pressure have been subject to recent investigation, especially in light of the decreased cost of genotyping technologies and increased research about schistosome molecular markers associated with PZQ resistance (Norton et al., 2010; Mendes et al., 2018; Gower et al., 2017; Doyle and Cotton, 2019; Berger et al., 2021; Summers et al., 2022). There have also been concerns that MDA would create a genetic bottleneck that selects for PZQ-resistant schistosomes (Norton et al., 2010; Rey et al., 2021). Genomic studies have reported genetic ramifications in *S. mansoni* worms following MDA and laboratory-induced resistance (Norton et al., 2010; Mendes et al., 2018; Gower et al., 2017). However, these studies are mainly aimed at investigating a limited number of molecular markers, and the vast number of unknown variables of genetic diversity makes the data difficult to attribute to the development of drug resistance (Coghlan et al., 2019; Doyle and Cotton, 2019; Berger et al., 2021). As a whole, genomic studies investigating PZQ resistance have found no long-term decrease in the genetic diversity of *S. mansoni* worms, even in ones that survived MDA (Gower et al., 2017; Faust et al., 2019).

### 3.3 Future research and vaccine

The WHO states the need for developing new drugs to co-administer with PZQ in case of resistance (World Health Organization, 2020). Several potential new compounds are PZQ derivatives, including sulphonamides, organometallics, and another agent with a minor structural variation to PZQ (Hess et al., 2015; Angeli et al., 2022; Xu et al., 2023). However, further testing and optimization is needed before such drugs become commercially available.

Because MDA alone is insufficient to eliminate schistosomiasis, the WHO also calls for the development of a schistosomiasis vaccine (World Health Organization, 2020). Recent advances in vaccine development increase the possibility of this goal being obtained. There are currently several schistosomiasis vaccines undergoing clinical testing (Molehin, 2020; Hotez and Bottazzi, 2023). Most are based on recombinant proteins and target *S. mansoni* (Zhang et al., 2020; Santini-Oliveira et al., 2022; Diemert et al., 2023). However, the task remains difficult due to the complex life cycle, host-evasion mechanisms, and hybridization between schistosome species (Fonseca et al., 2015). In addition, sustainable financing, uncertain manufacturer investment, and distribution issues remain considerable challenges. However, a schistosomiasis vaccine introduction in conjunction with MDA is a necessary factor in eliminating the disease, especially before any major sign of emerging schistosome resistance is detected (World Health Organization, 2023b).
